# An Investigation into the High Prevalence of Hepatitis B in a Rural Area of Kerala State, India: Hypothesis on* Chrysops* sp. (Diptera: Tabanidae) Transmission

**DOI:** 10.1155/2018/4612472

**Published:** 2018-06-10

**Authors:** Mariamma Kuriakose, Abraham M. Ittyachen

**Affiliations:** Department of Medicine, M.O.S.C. Medical College & Hospital, Kolenchery, Ernakulam District, Kerala State 682311, India

## Abstract

**Objective:**

Since 2005 there have been several reports of hepatitis B outbreak in the state of Kerala in southern India. Objective of this study was to analyze such outbreaks and to explore hypothesis pertaining the transmission mode.

**Methods:**

Retrospective observational study involving cases of acute hepatitis B acquired between 1 January 2015 and 31 December 2015 and their family members residing in Mazhuvanoor village in Ernakulam district of Kerala State in southern India.

**Results:**

59 houses were included in the survey. The number of patients diagnosed to have acute viral hepatitis B was 59. Majority (66.10%) were over 50 years old. There were no cases below the age of 15 years. All 59 patients claimed to have been bitten frequently by a fly which was identified as “deer fly” belonging to the genus* Chrysops*.

**Conclusion:**

Given the current understanding of mechanical transmission of pathogens in both humans and animals by insects belonging to the Tabanidae family which also includes* Chrysops*, it is plausible that the same mechanism may hold true for hepatitis B also. However this needs to be proven in further studies both at the laboratory level and at field studies.

## 1. Introduction

Hepatitis B infection is caused by a DNA (deoxyribonucleic acid) virus, the hepatitis B virus (HBV). HBV is the prototype member of the Hepadnaviridae family. Members of this family of viruses have a narrow host range and predominantly infect hepatocytes in their respective hosts [1]. HBV infection can be either acute or chronic and may range from asymptomatic infection or mild disease to severe or rarely fulminant hepatitis [2].

Acute hepatitis B is marked by acute inflammation and hepatocellular necrosis, with a case fatality rate of 0.5–1% [2]. Unlike its chronic variant acute hepatitis B is usually a self-limiting disease. Chronic hepatitis B infection is defined as persistent HBV infection with or without associated active viral replication and indication of hepatocellular injury [2]. Persistent HBV infection would mean the existence of hepatitis B surface antigen [HBsAg] in the blood or serum for longer than six months. Age plays an important role in chronic infection; risk of chronicity is more in neonates and young children than in infection that is acquired in adulthood. Risk of chronicity is 90% in neonates and 20-60% in children under the age of 5 years while it is less than 5% when acquired in adulthood [3, 4]. Around the world, majority of people who have chronic hepatitis B acquired the infection at birth or in early childhood.

Chronic HBV infection has a myriad presentation and outcome. In some people, it is inactive and does not lead to any significant liver disease (chronic carrier state). In others, it may gradually lead to fibrosis and ultimately end in cirrhosis and end-stage liver disease. In yet others it acts as a trigger for hepatocellular carcinoma (HCC) [5]. Longitudinal studies of untreated persons with chronic hepatitis B have shown that there is an 8–20% cumulative risk of developing cirrhosis over the next five years [3–7]. The annual incidence of hepatitis B-associated HCC ranges from <1% to 5% [8].

Age-specific HBsAg seroprevalence varies markedly by geographical region. Highest prevalence (>5%) is seen in the Pacific Islands, the Amazon Basin of South America, sub-Saharan Africa, East Asia, and some parts of the Balkan regions [9]. Around the world evidence of past or present infection is manifest in approximately 2 billion people [9]. About 240 million are chronic carriers of the HBV surface antigen (HBsAg) [9].

There is a serious dearth of data regarding the true prevalence of HBV in India. HBsAg positivity has been reported to range between 2% and 8% in most studies [10–12]. The most widely quoted figure of carrier rate in India is 4.7% with an estimated carrier population of 56.5 million [11]. Many of these studies are based on data from blood banks and may not truly reflect the national prevalence.

Kerala State is a province in the south-western part of peninsular India. Like the rest of the country data regarding the true prevalence of hepatitis B is deficient in this region also. Available studies are hospital based studies and not community based ones. In a five year retrospective analysis of viral hepatitis from our own institution, 6.35% were due to hepatitis B [13]. In another hospital based study from north Kerala, HBsAg positivity ranged from 0.71% to 4.49% [14]. Hospital based studies may not be representative of the community at large.

Since 2005 there have been several reports of hepatitis B outbreak in Kerala. Some of them have been reported in the media [15–17]. Many are anecdotal and many more may have gone unreported. These probably represent only the tip of the ice-berg. Only very few of these have been reported in medical literature [18]. Though the health authorities have made attempts to analyze these outbreaks, no consensual explanation was brought so far.

## 2. Methods

This is a community based study done in a rural area of Kerala State in the southern part of India. An unusually high number of hepatitis B cases was reported in the year 2015 from Mazhuvannoor village situated about four kilometers from our hospital by our own staff hailing from the same village. Local media reports also alluded to this.

**Figure 1:**
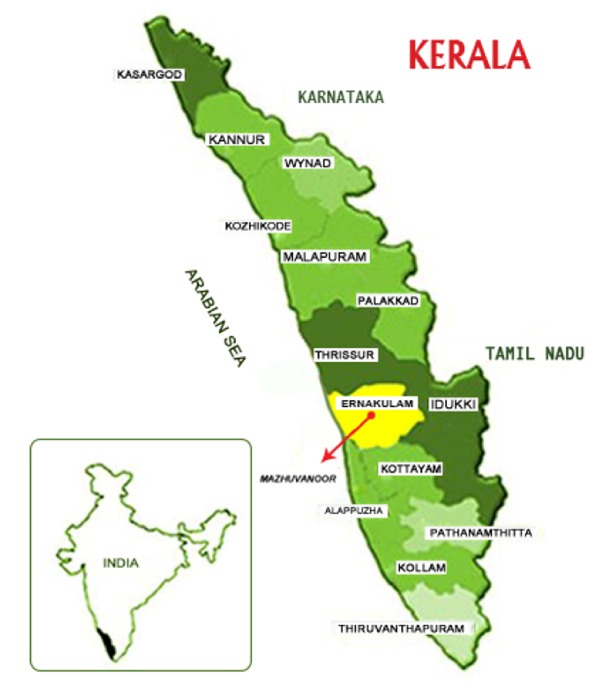
Mazhuvanoor village in Ernakulam district of central Kerala in southern India.

**Figure 2:**
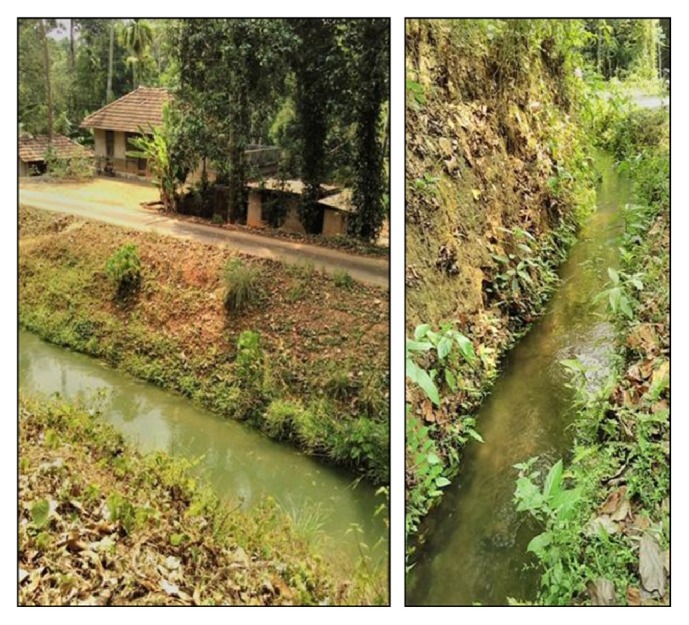
Irrigation canals.

Mazhuvanoor is a village located in the Kunnathunad area of Ernakulam district of central Kerala, not far from the high ranges of the Western Ghats (Figure 1). It has got a population of 14946 as per the population census of 2011 [19, 20]. The population is also highly literate with a literacy rate of 96.07% which is slightly more than 94.00% for the whole of Kerala State [20]. The village is divided into nineteen administrative units called wards. The economy is largely agrarian with rice, rubber, and pineapple being the main crops. The climate is humid and receives rainfall mainly in the monsoon season, southwest monsoon from June to August and the northeast monsoon from October to November. Besides there are also irrigation canals, big and small, which crisscross the area (Figure 2). With abundant supply of water, the land remains moist and sometimes water logged with dead and decaying vegetation. These areas are apt breeding grounds for many disease causing vectors with leptospirosis and dengue already being reported [21–23]. With a high density of population, people and cattle live in close proximity to these areas.

**Figure 3:**
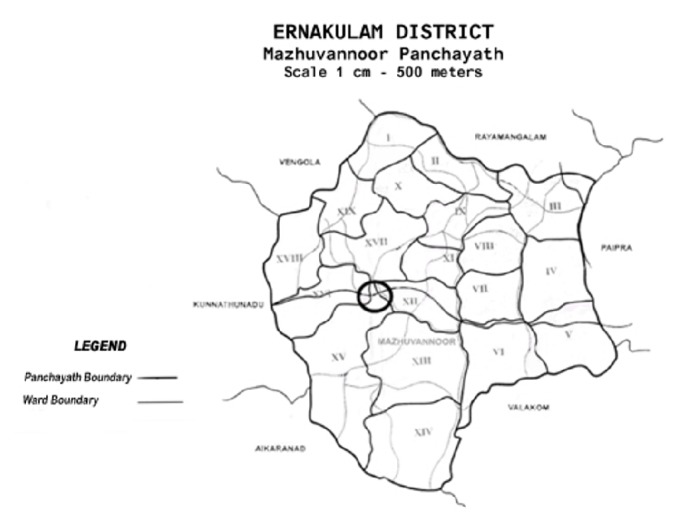
Study area in Mazhuvannoor village, in circle.

The study area included adjoining regions of wards 12, 15, and 16 spread over 3 square kilometers with 256 houses and a total population of 800. The actual participants in the study were however lesser than this. 59 houses were surveyed. In total 189 people were interviewed directly which included patients and their relatives living in the same house. The area covered was continuous with each other (Figure 3) but did not include the entire ward. Several visits were carried out in these aforementioned areas over a period of two months. Only clinical cases of acute hepatitis B that were proven by Elisa test and their family members were included in the study. No population survey was done for asymptomatic seropositivity. Patients who had acquired the infection between 1 January 2015 to 31 December 2015 were included in the study. Data was collected using a preset questionnaire with respect to age, sex, occupation, comorbidities, vaccination status, and known risk factors for acquiring hepatitis B. Anything unusual noted by the people prior to the onset of their illness was also enquired about. The vicinity of the houses in which the patients resided was also inspected. The people were sensitized as to the importance of the study and informed consent was taken from the participants. Ethics approval was obtained from the institutional review board of our institution.

## 3. Results

**Figure 4:**
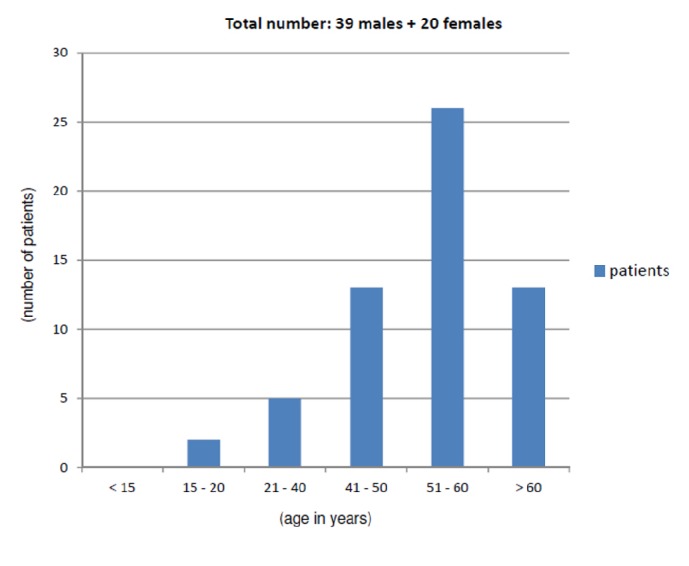
Number of patients.

59 houses were included in the survey and in total 189 people were directly interviewed. The number of patients diagnosed to have acute viral hepatitis B was 59. There were 39 (66.10%) male patients and 20 (33.90%) female patients. Majority were over 50 years old (66.10%) and were involved in small scale farming and animal rearing usually after retiring from a regular job. There were no cases below the age of 15 years (Figure 4). There were two families with two cases each, both husband and wife. None of the patients had received vaccination against hepatitis B. Maximum concentration of cases was found clustered in two housing colonies: a canal bund area and a marshy land area with a pig farm.

Of the 59 diagnosed cases of acute viral hepatitis B, 44 (74.58%) had one or the other of the conventional risk factors for hepatitis B transmission. 18 patients had injections and/or laboratory tests in the previous six months that involved the drawing of blood. The setting in which these were done could not immediately be verified. 9 patients used to make use of the services of the local barber for hair cutting and shaving. 11 patients in addition to the visit to the barber had some sort of injection and/or laboratory test. 3 patients had a dental procedure, 1 patient had upper gastrointestinal endoscopy, 1 patient had donated blood, and 1 patient had delivered in the hospital within the previous six months.

15 (25.42%) (8 females and 7 males) patients did not have any exposure according to the existing known methods of transmission of hepatitis B. All of them were elderly but otherwise healthy individuals who rarely ventured out of their village. None had any sexual exposure, injections or laboratory tests and dental or surgical procedures within six months prior to the hepatitis B infection.

All 59 (100%) patients including 44 in the conventional exposure group and the 15 with no known risk factors for hepatitis B claimed to have been bitten frequently by an unidentified fly. This fly they claimed to have about the same size as the common house fly with transparent wings and brown or black cross markings. This fly had recently made its appearance in their lives and was found around cow sheds and in grazing areas. It used to bite both people and animals and the bite was very painful and would bleed profusely. The fly was caught manually with the help of the local people. It was identified as “deer fly” belonging to the genus* Chrysops*.

## 4. Discussion

Since 2005 there have been sporadic outbreaks of hepatitis B in Kerala. Attempts to analyze these outbreaks have not led to any credible conclusion and these epidemics continue to smolder in spite of the best of efforts of the local health authorities. Mazhuvanoor is a village in central Kerala where such an outbreak occurred in 2015.

The study area was small, spread over just 3 square kilometers with 256 houses and a total population of 800. The actual participants in the study were even much lesser with 59 houses and 189 people. The sudden outbreak of this disease had attracted the attention of the media. With a high level of literacy, people were aware of the methods of transmission of hepatitis B and the consequences including cirrhosis and hepatocellular carcinoma. Due to this a stigma had crept into the population regarding this disease. As a result people were not always forthcoming regarding the presence of hepatitis B in their family. Barriers to screening for hepatitis B due to a traditional stigma attached to this disease has already been described in Asian communities [24, 25]. Also hepatitis B infection is subclinical in 70 percent of adults and 90 percent of children younger than five years [26, 27]. Hence we presume that the actual incidence of hepatitis B may even be higher.

Only patients above the age of 15 years had contracted hepatitis B. There were no reports of anyone below the age of 15 getting this disease. This is probably a successful manifestation of vaccination against hepatitis B received as part of the universal immunization program in childhood. However it could also be a consequence of the low prevalence of symptomatic acute cases in the young [4].

HBV is transmitted through percutaneous or parenteral contact with infected blood and body fluids and by sexual intercourse [28]. HBV does not cross intact skin or mucous membrane unless there are some breaks in this barrier. HBV also does not cross the placenta; hence it cannot infect the foetus unless there has been a break in the maternal-foetal barrier; this may occur during child birth [28]. Of the 59 patients who had acute hepatitis B, 44 had one or the other risk factor described for this disease. However there were no reports of anyone else who had used the services from the same source (hospital/laboratory tests/barber/dentist) contracting hepatitis B which in turn would make it highly unlikely that this 44 would have contracted the disease from these sources. And all these patients had history of frequent bites by the deer fly. The other 15 in the group were elderly folks who rarely ventured out of their village. They did not have any of the conventional risk factors described above for hepatitis B. All of them claimed to have been bitten by the fly.

**Figure 5:**
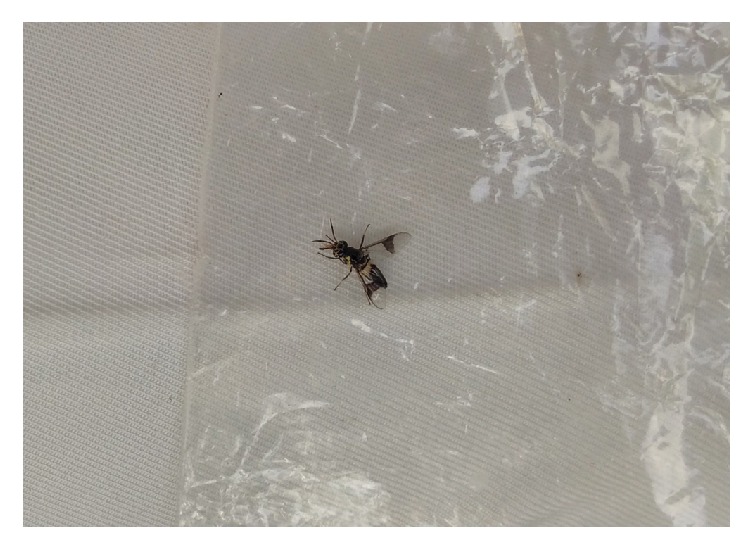
Actual specimen of “deer fly” trapped from the field.

The fly was identified as “deer fly”, belonging to the genus* Chrysops* (Figure 5). The genus* Chrysops* belongs to the order Diptera, family Tabanidae. The Tabanidae family contains some of the largest blood-sucking insects. Other than* Chrysops* the important genera in the Tabanidae family are* Haematopota *and* Tabanus *[29]. Tabanids (members of the Tabanidae family) are known for the pain their bite can cause and their size. They are more commonly known by local names which includes deerflies, mainly for* Chrysops*; horseflies, mainly for* Tabanus*; and clegs, mainly for* Haematopota*.

The earliest known description about tabanids in India is in 1798 [30]. Since then several species have been described [31]. However most of these are from a veterinary point of view. The role these insects have in the transmission of diseases to humans in India needs to be better elucidated. The geographical range of distribution of some of these insects is also not known. From an entomological point of view, why some species have appeared in hitherto unknown areas as was reported in this case also needs to be studied.

Tabanids can be pests of both livestock and humans. Since their bites are painful it often evokes a sharp response from the animal on which they are feeding. But being determined feeders they move from the disturbed animal to another animal to resume feeding. Because of this feeding nature and the “spongy” nature of the labella, the mouth part which they use for ingesting blood and which can hold up to 1 to 5 nanoliters of blood [32] makes them superb mechanical vectors of a number of disease causing agents [29]. Mechanical transmission as described above occurs when the disease agent is transmitted without amplification and development within the fly via contaminated blood on mouthparts (mechanical transmission of viruses, such as equine infectious anemia virus, vesicular stomatitis virus, protozoa, such as* Trypanosoma evansi* and* Besnoitia besnoiti*, and bacteria such as* Bacillus anthracis*,* Anaplasma marginale*,* Francisella tularensis*, and* Pasteurella multocida*) [33]. Transmission of disease agents can occur biologically also [34, 35]. Biological transmission occurs when the disease agent replicates or develops within the fly prior to transmission (Loa Loa) [33]. However Tabanids have never been shown to be biological vectors of viruses so far.

Mechanical transmission however has its limits [33]. Some minimal conditions require to be satisfied for successful mechanical transmission. They are as follows: (i) there should be high density of mechanical vectors, (ii) there should be high pathogenaemia in donors, (iii) there should be close contact between recipients and donors, and (iv) high receptivity and susceptibility should be present in a major population of potential recipients. Based on some of these factors a mathematical model of pathogen transmission by a defined insect population has been developed to evaluate the importance of mechanical transmission. This model permits us to replicate the evolution of pathogen prevalence under various predictive circumstances. It could also help us in control measures and could also be used to assess the risk of mechanical transmission under field conditions [36].

### 4.1. Possibility of Transmission of Hepatitis B by an Insect: A Proposed Hypothesis

**Figure 6:**
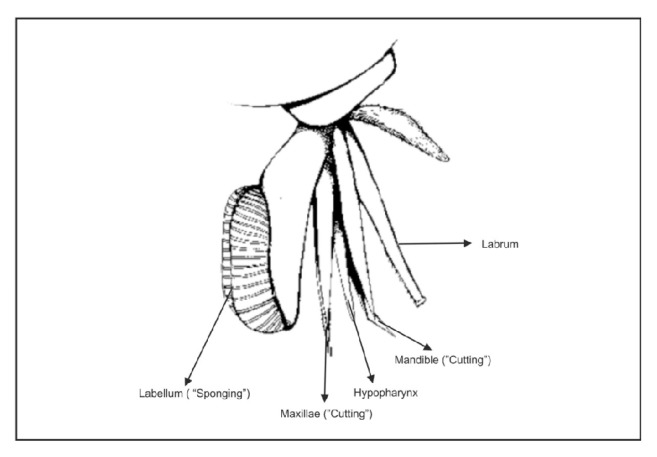
Mouth parts of the “deer fly”.

**Figure 7:**
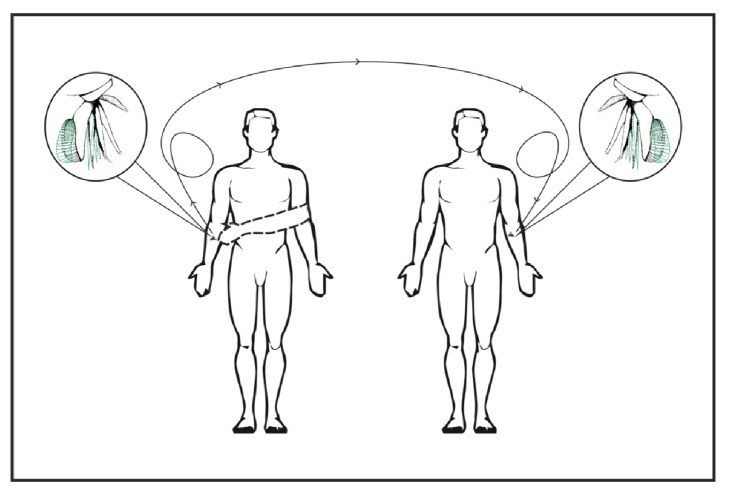
Postulated mechanical transmission of virus through contaminated mouth parts of the “deer fly”.

As already explained* Chrysops* flies are known to transmit disease causing agents mechanically [37]. These flies inflict deep wounds with their mandibles and maxillae that penetrate the skin in a scissor-like action. Anticoagulants in the saliva are pumped into the wound and the blood is ingested through the sponging labella (Figure 6). Pathogens may be transmitted from flies that are disturbed while feeding on one animal and begin feeding on another. The same process may hold true for humans also. When a fly bites a person who is infected with hepatitis B and feeds on his blood the pain naturally elicits an intense response from the person. This would disturb the fly which may fly off and attack another person and in the process transmit the virus through its contaminated mouth parts (Figure 7). Hepatitis B is highly contagious. Even a minimal amount of blood is known to transmit this infection [38, 39]. It can survive outside the body for up to 7 days and hence can also be transmitted via contaminated objects [40]. Even in health care workers the risk of transmission is 6 to 30% following a percutaneous exposure [41]. And it is also known that hepatitis B is 50 to 100 times more infectious than HIV (Human Immunodeficiency Virus) [42].

Since it is known that hepatitis B can survive outside the environment for up to 1 week we can hypothesize that an insect might stay infective from a blood meal to another, at several days interval. This aspect combined with the presence of an infected subject (donor) with a high viremia who stays close to another subject (recipient) facilitates rapid transmission of the virus.

## 5. Conclusion

A high incidence of hepatitis B in such a small population and in an even smaller area, not to mention the number of undisclosed/subclinical cases in the same community, calls for more research especially when the only clue available is novel, “bitten by the deer fly”.

Given the current understanding of mechanical transmission of several pathogens in both humans and animals by insects belonging to the Tabanidae family which also includes* Chrysops*, it is plausible that the same mechanism may hold true for hepatitis B also. However this needs to be proven in further studies at both the laboratory level and field studies. Laboratory studies could include transmission of the virus under controlled conditions using animal models. In the field, investigation of an epidemic should try to zero on the index case and establish whether the same genotype is involved in all the cases. Confirmation of such a possibility could have major ramifications including transmission of other blood borne pathogens. In the meantime universal immunization against hepatitis B may be the only solution.

## Data Availability

The data used to support the findings of this study are included within the article.
